# Systematic review and meta-analysis of the association between young maternal age and fetal abnormalities

**DOI:** 10.1038/s41598-024-74015-1

**Published:** 2024-09-29

**Authors:** Jakub Staniczek, Maisa Manasar-Dyrbuś, Magda Rybak-Krzyszkowska, Adrianna Kondracka, Dominika Orszulak, Kacper Niziński, Marcin Sadłocha, Agnieszka Drosdzol-Cop, Rafał Stojko, Miriam Illa

**Affiliations:** 1 Department of Gynecology, Obstetrics and Gynecological Oncology, Medical University of Silesia, Katowice, 40-211 Poland; 2 Department of Obstetrics and Perinatology, University Hospital, Krakow, 30-688 Poland; 3 Department of Obstetrics and Pathology of Pregnancy, Medical University of Lublin, Lublin, 20-059 Poland; 4 BCNatal | Fetal Medicine Research Center (Hospital Clínic and Hospital Sant Joan de Déu), Universitat de Barcelona, Barcelona, 08028 Spain

**Keywords:** Congenital anomalies, Fetal abnormalities, Young maternal age, Adolescent mothers, Paediatrics, Public health, Health care, Medical research, Risk factors

## Abstract

Fetal anomalies among young women and adolescents pose major public health concerns. This systematic review aims to elucidate the relationship between young maternal age and the incidence of fetal abnormalities. According to the systematic review and meta-analysis PRISMA protocol, cohort, cross-sectional and case–control studies were scrutinized to include 80,393,450 participants across diverse regions. The meta-analysis utilized Odds Ratios (OR) as the effect measure, adopting a random-effects model. The screening process involved 157 selected and verified manuscripts, which ultimately resulted in the final inclusion of 20 studied in the meta-analysis. The criterion for young maternal age was the age of ≤ 20 years. The meta-analysis revealed a pooled OR of 0.93 (95% CI: 0.82–1.05, *p* = 0.252), indicating no statistically significant association between young maternal age (≤ 20 years) and fetal anomalies. However, considerable heterogeneity (I² = 96.21%) was noted, prompting the use of a random-effects model to derive the reported results. The meta-analysis did not find statistically significant differences in the occurrence of congenital anomalies in fetuses of younger women than in overall population. Although due to significant heterogeneity of the analyzed studies, and a risk of bias, caution should be exercised when interpreting the results, further investigation may be warranted to understand the relationship between maternal age and risk of fetal anomalies. Nevertheless, the study shows significant differences, which diminish in collective analysis, suggests that factors beyond age may be influential. Specifically, the limited access to or quality of healthcare in certain regions could be a more critical factor than maternal age itself.

## Introduction

Fetal abnormalities are some of the leading health problems that affect young women and their children. The World Health Organization attributes approximately 240,000 deaths globally to congenital fetal abnormalities during the first four weeks of life. These fatalities are particularly common among fetuses with neural tube defects^1^.

Son et al. estimate that 10% of stillbirths arise from fetal anomalies, which may also be common in live births^2^. According to the World Health Organization, 170,000 children aged between 1 month and 5 years die of birth-related anomalies. Adolescents and young women from poor neighborhoods are prone to giving birth to children with congenital fetal anomalies due to a lack of access to vaccination facilities, insufficient intake of folic acid, and inadequate care before and during pregnancy^1^. Young women who are exposed to maternal infections are vulnerable to congenital fetal anomalies. Serunjogi et al. also observed that regions with high adolescent pregnancy rates record many cases of fetal abnormalities. Teenage mothers are more likely to experience adverse birth outcomes than females aged 20 to 34 years^3^. The 63rd World Health Assembly resolved to encourage nations to formulate country-specific policies for adopting fetal anomalies surveillance systems and prevention of such conditions. Therefore, exploring the relationship between maternal age and congenital fetal abnormalities is crucial in developing preventive strategies and assessing the factors that contribute to this health crisis.

More specifically, healthcare professionals are continually creating awareness about the adverse effects of congenital fetal anomalies. In response, many researchers are exploring mechanisms for preventing, diagnosing, and implementing screening programs to facilitate continuous support and care for affected children. Studies have attempted to examine the adverse impacts of congenital anomalies^4^. However, such investigations focus primarily on the methods of preventing, diagnosing, and managing the condition. Consequently, major literature gaps still exist on the effects of young maternal age and fetal abnormalities. Therefore, this study aims to seal these research gaps by using a systematic review and meta-analysis methods to explore the correlation between young maternal age and fetal anomalies.

## Hypothesis

For the purposes of this publication, the definition of adolescent pregnancy adopted is that of the World Health Organization, which is characterized by a pregnant woman aged between 10 and 19 years. Additionally, in accordance with the Polish Central Statistical Office, pregnancies are categorized as ‘19 years and under’^4^. Congenital fetal abnormalities are defined as structural or functional anomalies that occur during intrauterine life and can be identified prenatally, or at birth. The principal hypothesis guiding this systematic review and meta-analysis suggests that adolescent pregnancy (defined herein as gestation in females aged ≤ 20 years) is associated with an elevated incidence of congenital fetal anomalies in comparison to pregnancies in females aged over 20 years. This hypothesis is predicated on the belief that a younger maternal age may contribute to a higher rate of adverse pregnancy outcomes, including fetal abnormalities, relative to older women.

## Methods

The PRISMA model was adopted to guide the process of assessing the quality of studies and summarizing the evidence^5^. This procedure involved synthesizing the pieces of evidence gathered by tabulating study characteristics, quality, and impact. Additionally, statistical tools were used to compare the studies and combine their effects using a meta-analysis. After exploring the heterogeneity of the data of each source, the trustworthiness of the overall summary was assessed. All the recommendations and findings were also graded based on the strengths and weaknesses of the evidence that they provided. To extract as many relevant sources as possible, a broad range of cohort, cross-sectional, case–control studies from the medical databases was searched. After the studies of acceptable research designs were identified and selected, their risks of biases were evaluated to determine the quality of the evidence in refined ways.

### Eligibility criteria

The inclusion criteria of this study were focused on English-language, peer-reviewed journal articles that specifically utilized cohort, cross-sectional and case–control study methodologies. The exclusion criteria encompassed sources that did not employ cohort, cross-sectional or case–control studies and study materials with lower internal and external validity. Moreover, case reports, conference abstracts and letters to the editors along with review articles or guidelines were excluded.

### Information sources

The databases that were searched for all relevant materials for this systematic review and meta-analysis included EMBASE, PubMed, Cochrane, and Web of Science. Institutional review board, as well as the ethical committee approval were not obtained, as the systematic review and meta-analysis involved the retrospective analyses of de-identifying studies that had already been published.

### Search strategy

The literature searches were conducted from June 1, 2023, to November 31, 2023. The inclusive dates for the included articles range from January 2010 to November 2023.

Keywords and phrases were used by employing synonyms that were related to the research topic, such as fetal anomalies, congenital fetal abnormalities, and congenital complications. Additionally, the concepts that were relevant to the research topic were searched on databases, and the results were scanned for alternative words and phrases.

Search string: ((adolescent pregnancy [MeSH Terms]) OR (adolescent pregnancy [Title/Abstract]) OR (adolescent pregnancies[MeSH Terms]) OR (adolescent pregnancies [Title/Abstract] ) OR (adolescence, pregnancy in[MeSH Terms]) OR (Adolescent Mothers[MeSH Terms]) OR (Adolescent Mothers [Title/Abstract]) OR (young maternal age[Title/Abstract]) OR (teenage mother [Title/Abstract])) AND ((congenital abnormalities [MeSH Terms]) OR (congenital abnormalities [Title/Abstract]) OR (birth defect [Title/Abstract]) OR (fetal anomalies [Title/Abstract]))

### Selection process

In this systematic review, we implemented a two-phase selection process. Initially, we screened titles and abstracts to ensure they met our predefined inclusion criteria, effectively filtering out unrelated studies. Following this, we conducted an in-depth examination of the full texts of the preliminarily selected articles, focusing specifically on evaluating their external and internal validity.

### Data collection process

The data collection process began with the development of a data extraction template, which was tailored to uniformly capture all essential information from each selected study. This template included details such as the study’s author, publication year, methodology, sample size, and key findings or outcomes. All relevant data were meticulously extracted from each study, ensuring consistency and accuracy in recording (Table 2). This step often involved cross-checking to mitigate errors and bias. The data collection process also entailed comparing the appropriate outcome measures and exploring the degree of between-study inconsistencies to avoid biases. This stage of the collection process was critical in pooling the data and calculating the summary measures and confidence interval. The collected data was then compiled and organized for analysis, providing a comprehensive dataset that underpinned the review’s findings and conclusions (Table 3).

### Data items

The data outcomes were operationalized into measurable units such as prevalence, incident, and mortality rates arising from congenital fetal anomalies (Table 4).

### Reporting bias assessment

The assessment of study quality was carried out by two separate researchers. For evaluating studies, the Newcastle–Ottawa scale (NOS) was employed^6^. In the case of randomized studies, their quality was appraised using the revised Cochrane risk of bias tool 2.0^7^. Any differences in opinion among the authors were thoroughly discussed and resolved. The quality of most studies was rated “good.” Detailed assessments are presented in Table 5.

### Synthesis method

The collected data was synthesized using meta-analysis, aggregating the results from the selected studies to form a comprehensive understanding of the research question. This process involved quantifying effect sizes from each study and pooling them using appropriate statistical models. It also included evaluations for heterogeneity and potential biases, ensuring the reliability and validity of the synthesized findings. This method effectively combined individual study results into a significant and generalizable conclusion. Due to retrospective character of the included studies, and the higher risk of bias inherent with further subdivision of the population. Subgroup analysis was conducted, considering the region where the study was performed and the type of anomaly depending on the system they affect. Owing to the availability of data in the primary studies, the analysis based on the type of anomaly was conducted as follows: the proportion of this type of defect was examined, and a logarithmic transformation was applied to normalize its distribution.

## Results

### Study selection

**Fig. 1:**
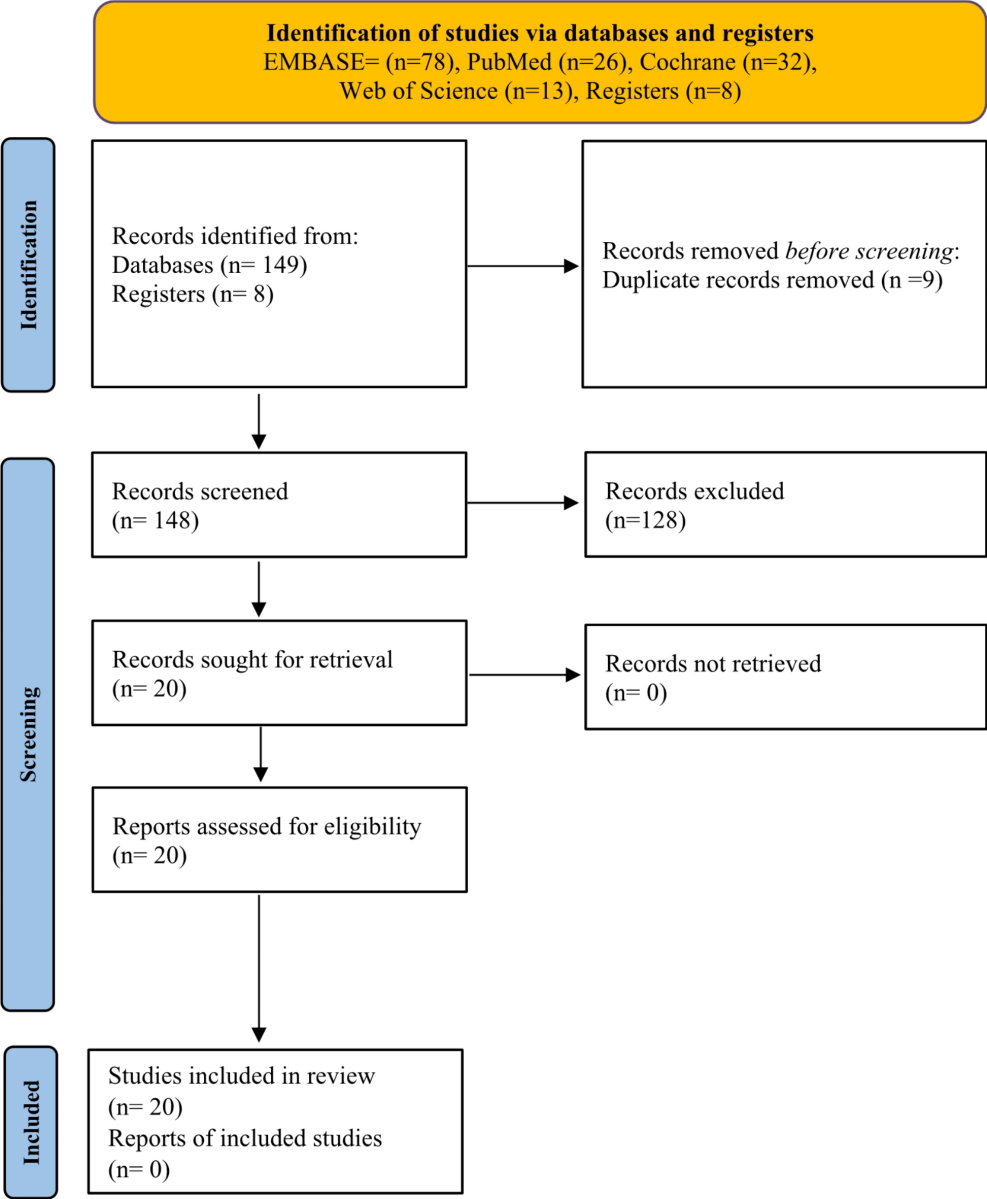
A PRISMA Flow chart of the study inclusion process.

Two reviewers performed the process of selecting the studies to incorporate into this analysis. This procedure involved using search strategies to access and filter studies from four major databases: EMBASE, PubMed, Cochrane, and Web of Science. Additionally, studies that reported events related to fetal anomalies among teenage mothers were incorporated into the review process. These anomalies could have encompassed a range of conditions, such as neural tube defects, abdominal wall defects, heart defects, and other structural or functional anomalies identified through prenatal screening, diagnostic tests, or at birth. No restrictions were employed for research designs, languages, and geographical locations of each study reviewed. Some of the details that were retrieved from the studies included in the systematic review and meta-analysis are first authors ‘last names, year of publication, sample size, correlation, effect direction, standard error, and p-value. The number of studies selected from each database included 78 (EMBASE), 26 (PubMed), 2 (Cochrane), and 43 (Web of Science). Out of these articles, after deletion of duplicates, only 20 were found to be eligible for conducting the systematic review and meta-analysis. (Fig. 1)

### Study characteristics and results of individual studies

Analyzing the collection of studies on congenital fetal anomalies, it’s evident that they incorporate a diverse range of methodologies and cover various geographical regions. The methodologies include 2 case-control studies, 6 cohort studies, 10 cross-sectional studies, and 2 nested case-control studies.

Geographically, these studies span across Ethiopia, Europe, the USA, China, Thailand, Saudi Arabia, Argentina, Ireland, Finland, and Taiwan. The time frame of the studies ranges from 2014 to 2021, encompassing a wide array of participant numbers, from a few thousand individuals to several million, with a total 80,393,450 participants across all studies. This diverse set of studies, both in terms of methodology and geographical representation, offers a comprehensive view of the prevalence and risk factors associated with congenital fetal anomalies. (Table 1). Additionally, Table 2 includes confounders and covariates of the publications that were used in the systematic review.

Table 3 presents a detailed analysis of various congenital fetal anomalies, highlighting the number of patients affected by specific anomalies and identifying the groups with the highest prevalence. The studies encompass a range of anomalies including neural tube defects, hypospadias, congenital heart disease, microcephaly, anotia/microtia, congenital limb defects, cleft lip/palate, holoprosencephaly, omphalocele, diaphragmatic hernia, gastroschisis, and others. Some studies focused on a single anomaly, like Bergman’s^9^ on hypospadias, Best’s^10,11^ on congenital heart disease, and Cragan’s^9^ on microcephaly. Others examined multiple anomalies, like Soressa Abebe’s^8^ and Jiang’s studie^16^, which reported on a variety of conditions including neural tube defects, congenital heart defects, and cleft lip/palate. Collectively, these studies provide a comprehensive picture of the prevalence of various congenital fetal anomalies, contributing valuable data to the field. The table, rich in detail, offers an essential reference for understanding the specific prevalence rates and the most affected groups for each anomaly (Tables 3 and 4).

**Table 1:** Study characteristics.

First author; Year	Analysed period	Study type	Number of participants	Number of participants with fetal congenital anomaly	Region	Prevalence of congenital fetal abnormalities and odd ratio
Soressa Abebe 2021^8^	2016–2018	Case–control	35,080	251	Ethiopia	OR for age ≤ 20 years vs. > 20 years: 1.29 (95% CI 0.93–1.78) Prevalence: ≤20 years: 25.6%, > 20 years: 21.1%
Bergman 2015^9^	2001–2010	Cross-sectional	5,871,855	10,929	Europe	RR 1.14 (95% Cl 1.00-1.24) Prevalence: <20 years: 0.20%; >20 years 0.17%
Best 2014^10^	1980–2009	Cross-sectional	12,146,210	1322	Europe	RR: 0.96 (95% CI 0.70–1.29)
Best 2016^11^	1998–2013	Cross-sectional	499,826	4024	England	RR: 0.99 (95% CI 0.89–1.10)
Cragan 2016^12^	2009–2013	Cross-sectional	11,110,665	9678	USA	OR: 1.36 (95% CI 1.27–1.44) Prevalence ≤ 20 years: 0.11%, > 20 years: 0.083%
Deng 2015^13^	1996–2007	Cross-sectional	6,308,594	1933	China	OR: 0.61 (95% CI 0.30–1.22) Prevalence ≤ 20 years: 0.024%, > 20 years: 0.03%
Jaruratanasirikul 2016^14^	2009–2013	Cross-sectional	186,393	424	Thailand	OR: 0.62 (95% CI 0.43–0.89) Prevalence ≤ 20 years: 0.15% >20 years: 0.24%
Jaruratanasirikul 2016b^15^	2009–2013	Cross-sectional	186,393	269	Thailand	OR: 0.62 (95% CI 0.69–1.44) Prevalence ≤ 20 years: 0.14% >20 years: 0.14%
Jiang 2020^16^	2013–2017	Cohort	713,037	16,546	China	OR: 1.04 (95% CI 0.95–1.06) Prevalence ≤ 20 years: 2.42% >20 years: 2.32%
Kurdi 2019^17^	2010–2013	Nested case–control	28,646	1179	Saudi Arabia	OR: 0.53 (95% CI 0.32–0.86)
Ling 2019^18^	2007–2014	Cross-sectional	13,284,142	1222	China	OR: 1.02 (95% CI 0.70–1.50) Prevalence: ≤20 years: 0.01%, > 20 years: 0.01%
Marshall 2015^19^	1995–2005	Cohort	12,006,912	2308	USA	OR: 1.15 (95% CI 1.03–1.32) Prevalence: ≤20 years: 0.02%, > 20 years: 0.02%
Mburia-Mwalili 2015^20^	2006–2011	Cohort	124,341	4641	USA	OR: 0.74 (95% CI 0.62–0.89) Prevalence: ≤20 years: 2.81%, > 20 years: 3.76%
McGivern 2014^21^	1980–2009	Cross-sectional	12,155,491	3373	Europe	OR: 1.01 (95% CI 0.62–0.89) Prevalence: ≤20 years: 0.022%, > 20 years: 0.02%
Parker 2017^22^	1987–2012	Nested case–control	1,118	292	Finland	OR: 1.12 (95% CI 0.79–1.59)
Pawluk 2014^23^	1992–2001	Case–control	17,130	3786	Argentina	OR: 0.90 (95% CI 0.83–0.98)
Vaughan 2013^24^	2000–2011	Cohort	36,916	1172	Ireland	OR: 0.99 (95% CI 0.82–1.20) Prevalence: ≤20 years: 3.17%, > 20 years: 3.14%
Weng 2014^25^	2001–2010	Cohort	2,123,751	No data	Taiwan	OR: 1.42 (95% CI 1.39–1.44)
Xie 2018 ^26^	2012–2016	Cross-sectional	673,060	6289	China	OR: 0.66 (95% CI 0.52–0.83) Prevalence: ≤20 years: 0.62%, > 20 years: 0.94%
Xie 2021^27^	2016–2019	Cross-sectional	2,883,890	3181	China	OR: 0.21 (95% CI 0.14–0.33) Prevalence: ≤20 years: 0.02%, > 20 years: 0.11%

**Table 2:** Confounders and covariates of the publications that were used in the systematic review.

First author, Year	Confounders	Covariates
Soressa Abebe 2021^8^	Maternal age, socioeconomic status, environmental factors, healthcare access, maternal health conditions.	Gender of the newborn, birth weight, prenatal care, maternal education, maternal nutrition.
Bergman 2015^9^	Geographic location, ethnicity, socioeconomic status, environmental exposures, genetic factors.	Maternal age, paternal age, parity, birth weight, gestational age.
Best 2014^10^	Genetic predispositions, geographic variations, socioeconomic factors, environmental exposures.	Maternal age, infant sex, birth weight, gestational age, parental occupation
Best 2016^11^	Maternal health conditions, genetic predispositions, environmental exposures, socioeconomic status.	Maternal age, maternal smoking, alcohol consumption, prenatal care, family history of heart disease.
Cragan 2016^12^	Zika virus exposure, maternal infections, genetic factors, environmental toxins.	Maternal age, maternal health conditions, socioeconomic status, prenatal care, race/ethnicity.
Deng 2015^13^	Environmental factors, genetic predispositions, maternal nutrition, healthcare access.	Maternal age, infant sex, birth weight, prenatal care, region of residence.
Jaruratanasirikul 2016^14^	Environmental exposures, genetic factors, maternal health conditions, socioeconomic status.	Maternal age, infant sex, birth weight, prenatal care, parental occupation.
Jaruratanasirikul 2016b^15^	Genetic factors, environmental exposures, maternal health conditions, socioeconomic status.	Maternal age, infant sex, birth weight, prenatal care, parental occupation.
Jiang 2020^16^	Healthcare access, environmental exposures, maternal health conditions, genetic factors.	Maternal age, preconception health status, socioeconomic status, prenatal care, parental education.
Kurdi 2019^17^	Genetic predispositions, environmental exposures, maternal health conditions, healthcare access.	Maternal age, infant sex, birth weight, gestational age, parental consanguinity.
Ling 2019^18^	Genetic factors, environmental exposures, maternal infections, healthcare access.	Maternal age, prenatal care, socioeconomic status, infant sex, birth weight.
Marshall 2015^19^	Genetic predispositions, environmental exposures, maternal health conditions, socioeconomic status.	Maternal age, prenatal care, birth weight, gestational age, race/ethnicity.
Mburia-Mwalili 2015^20^	Maternal health conditions, socioeconomic status, environmental exposures, genetic factors.	Maternal age, interpregnancy interval, prenatal care, birth weight, maternal education.
McGivern 2014^21^	Genetic predispositions, environmental exposures, maternal health conditions, healthcare access.	Maternal age, infant sex, birth weight, gestational age, prenatal care.
Parker 2017^22^	Maternal infections, genetic predispositions, socioeconomic status, environmental exposures.	Maternal age, prenatal care, maternal health conditions, birth weight, race/ethnicity.
Pawluk 2014^23^	Socioeconomic status, environmental exposures, healthcare access, genetic factors.	Maternal age, prenatal care, maternal education, birth weight, infant sex.
Vaughan 2013^24^	Maternal health conditions, socioeconomic status, environmental exposures, healthcare access.	Maternal age, prenatal care, birth weight, gestational age, parity.
Weng 2014^25^	Maternal health conditions, socioeconomic status, environmental exposures, genetic factors.	Maternal age, prenatal care, birth weight, gestational age, parity.
Xie 2018^26^	Genetic factors, environmental exposures, maternal health conditions, socioeconomic status.	Maternal age, infant sex, birth weight, prenatal care, region of residence.
Xie 2021^27^	Genetic predispositions, environmental exposures, maternal health conditions, healthcare access.	Maternal age, prenatal care, socioeconomic status, birth weight, infant sex

**Table 3:** Most common congenital fetal anomalies characteristics.

First author, Year	Anomaly of interest in the analysis	Anomaly groups with highest prevalence
Soressa Abebe 2021^8^	Any congenital fetal anomaly	Neural tube defects: 15.55% Musculoskeletal defects: 4.04% Gastrointestinal defects: 1.41% Urogenital defects: 0.62% Congenital heart defects: 0.09% Genetic disorders: 0.35%
Bergman 2015^9^	Hypospadias	Hypospadias: 0.19%
Best 2014^10^	Hirschsprung’s Disease	Hirschsprung’s Disease: 0.01%
Best 2016^11^	Congenital Heart Disease	Congenital heart defects: 0.81%
Cragan 2016^12^	Microcephaly	Microcephaly: 0.087%
Deng 2015^13^	Anotia/Microtia	Anotia/Microtia: 0.03%
Jaruratanasirikul 2016^14^	Congenital limb defects	Congenital limb defects: 0.23%
Jaruratanasirikul 2016b^15^	Cleft lip/palate	Cleft lip/palate: 0.14%
Jiang 2020^16^	Any congenital fetal anomaly	Neural tube defects: 0.08% Cleft lip/palate: 0.15% Anotia/microtia: 0.05% Hypospadias: 0.04% Congenital limb defects: 0.40% Gastroschisis: 0.03% Congenital heart defects: 0.61%
Kurdi 2019^17^	Any congenital fetal anomaly	Neural tube defects: 0.19% Anotia/microtia: 0.02% Congenital heart defects: 1.48%
Ling 2019^18^	Holoprosencephaly	Holoprosencephaly: 0.01%
Marshall 2015^19^	Omphalocele	Omphalocele: 0.02%
Mburia-Mwalili 2015^20^	Any congenital fetal anomaly	Congenital heart disease: 1.44% Cleft lip/palate: 0.08% Pyloric stenosis: 0.11% Urogenital defects: 0.40%
McGivern 2014^21^	Diaphragmatic hernia	Diaphragmatic hernia: 0.03%
Parker 2017^22^	Gastroschisis	Gastroschisis: 26%
Pawluk 2014^23^	Any congenital fetal anomaly	Omphalocele: 0.26% Gastroschisis: 0.57% Neural tube defects: 5.79% Microtia: 0.92% Cleft lip/palate: 3.60% Gastrointestinal defects: 1.00% Hypospadias: 0.93% Congenital heart defects: 3.14% Congenital limb defects: 3.91%
Vaughan 2013^24^	Any congenital fetal anomaly	Congenital abnormalities (any): 4.4%
Weng 2014^25^	Any congenital fetal anomaly	Congenital abnormalities (any): 1.42%
Xie 2018^26^	Congenital heart diseases	Congenital heart diseases: 0.93%
Xie 2021^27^	Any congenital fetal anomaly	Congenital heart defects: 4.62% Cleft lip/plate: 1.22% Congenital limb defects: 1.92% Urogenital defects: 0.69%

**Table 4:** Congenital fetal anomalies divided into groups of anomalies depending on the system they affect.

First author, Year	Anomaly groups with highest prevalence
Heart defects	
Soressa Abebe 2021^8^	Congenital heart defects: 0.09%
Best 2016^11^	Congenital heart defects: 0.81%
Jiang 2020^16^	Congenital heart defects: 0.61%
Kurdi 2019^17^	Congenital heart defects: 1.48%
Mburia-Mwalili 2015^20^	Congenital heart disease: 1.44%
Pawluk 2014^23^	Congenital heart defects: 3.14%
Xie 2018^26^	Congenital heart diseases: 0.93%
Xie 2021^27^	Congenital heart defects: 4.62%
Gastrointestinal defects	
Soressa Abebe 2021^8^	Gastrointestinal defects: 1.41%
Best 2014^10^	Hirschsprung’s Disease 4.1%
Jiang 2020^16^	Gastroschisis: 0.03%
Marshall 2015^19^	Omphalocele: 0.02%
Mburia-Mwalili 2015^20^	Pyloric stenosis: 0.11%
Parker 2017^22^	Gastroschisis: 26%
Pawluk 2014^23^	Gastroschisis: 0.57%
Pawluk 2014^23^	Omphalocele: 0.26%
Pawluk 2014^23^	Gastrointestinal defects: 1.00%
Neural tube defects	
Soressa Abebe 2021^8^	Neural tube defects: 15.55%
Jiang 2020^16^	Neural tube defects: 0.08%
Kurdi 2019^17^	Neural tube defects: 0.19%
Pawluk 2014^23^	Neural tube defects: 5.79%
Urogenital defects	
Soressa Abebe 2021^8^	Urogenital defects: 0.62%
Bergman 2015^9^	Hypospadias: 0.20%
Jiang 2020^16^	Hypospadias: 0.04%
Mburia-Mwalili 2015^20^	Urogenital defects: 0.40%
Pawluk 2014^23^	Hypospadias: 0.93%
Xie 2021^27^	Urogenital defects: 0.69%
Limb defects	
Jaruratanasirikul 2016^14^	Congenital limb defects: 0.23%
Jiang 2020^16^	Congenital limb defects: 0.40%
Mburia-Mwalili 2015^20^	Congenital limb defects: 3.91%
Face and head defects	
Cragan 2016^12^	Microcephaly: 0.09
Deng 2015^13^	Anotia/Microtia: 0.03%
Jaruratanasirikul 2016b^15^	Cleft lip/palate: 0.14%
Jiang 2020^16^	Cleft lip/palate: 0.15%
Jiang 2020^16^	Anotia/microtia: 0.05%
Kurdi 2019^17^	Anotia/microtia: 0.02%
Mburia-Mwalili 2015^20^	Cleft lip/palate: 0.08%
Pawluk 2014^23^	Microtia: 0.92%
Pawluk 2014^23^	Cleft lip/palate: 3.60%

### Risk of bias in studies

For the assessment of the quality of 20 of the included studies, we used Newcastle—Ottawa scale (NOS). In the cohort studies, the scores range from 6 to 9, with the majority rated as ‘Good’ (scores of 7 or higher), except for Vaughan 2013^24^ and Weng 2014^25^, which are rated ‘Fair’ and ‘Poor’, respectively. This indicates that higher scores in ‘Selection’ and ‘Exposure’ are pivotal for a ‘Good’ rating. The case-control studies show a score range of 5 to 8. Most are rated ‘Good’, but Pawluk^23^ stands out as ‘Poor’, primarily due to a zero in ‘Exposure’, highlighting the significant impact of low scores in any single category. The cross-sectional studies present a higher range of scores, from 7 to 10, predominantly rated as ‘Good’, with a couple of ‘Fair’ ratings for Xie 2018 and Xie 2021^26,27^. These studies generally exhibit high scores in ‘Selection’ and ‘Exposure’, with ‘Comparability’ showing less variation, usually scoring 1 or 2. (Table 5)

Comparing across study types, cross-sectional studies tend to have higher scores than cohort and case-control studies, possibly indicating more rigorous selection and exposure criteria. A consistent observation across all study types is that ‘Good’ ratings are common, but the criteria for achieving this rating can vary. Notably, any study receiving a zero in any category tends to be rated lower overall, as evidenced in Weng 2014^25^ (Cohort) and Pawluk 2014^23^ (Case-Control). (Table 5)

**Table 5:** Newcastle—Ottawa quality assessment scale for observational studies.

Study	Selection	Comparability	Exposure	Total Score	Assessment
Cohort studies (MAX. 9)					
Jiang 2020^16^	3	2	2	7	Good
Marshall 2015^19^	3	2	2	7	Good
Mburia-Mwalili 2015^20^	4	2	3	9	Good
Vaughan 2013^24^	2	2	2	6	Fair
Weng 2014^25^	4	0	2	7	Poor
Case-control studies (MAX. 9)					
Soressa Abebe 2021^8^	3	1	3	7	Good
Kurdi 2019^17^	4	1	3	8	Good
Parker 2017^22^	3	1	3	7	Good
Pawluk 2014^23^	4	1	0	5	Poor
Cross-sectional studies (MAX. 10)					
Bergman 2015^9^	4	2	3	9	Good
Best 2014^10^	5	1	3	9	Good
Best 2016^11^	5	1	3	9	Good
Cragan 2016^12^	5	2	3	10	Good
Deng 2015^13^	5	2	3	10	Good
Jaruratanasirikul 2016^14^	5	1	3	9	Good
Jaruratanasirikul 2016b^15^	5	1	3	9	Good
Ling 2019^18^	4	2	3	9	Good
McGivern 2014^21^	5	1	3	9	Good
Xie 2018^26^	3	1	3	7	Fair
Xie 2021^27^	4	1	3	7	Fair

### Meta-analysis

**Fig. 2:**
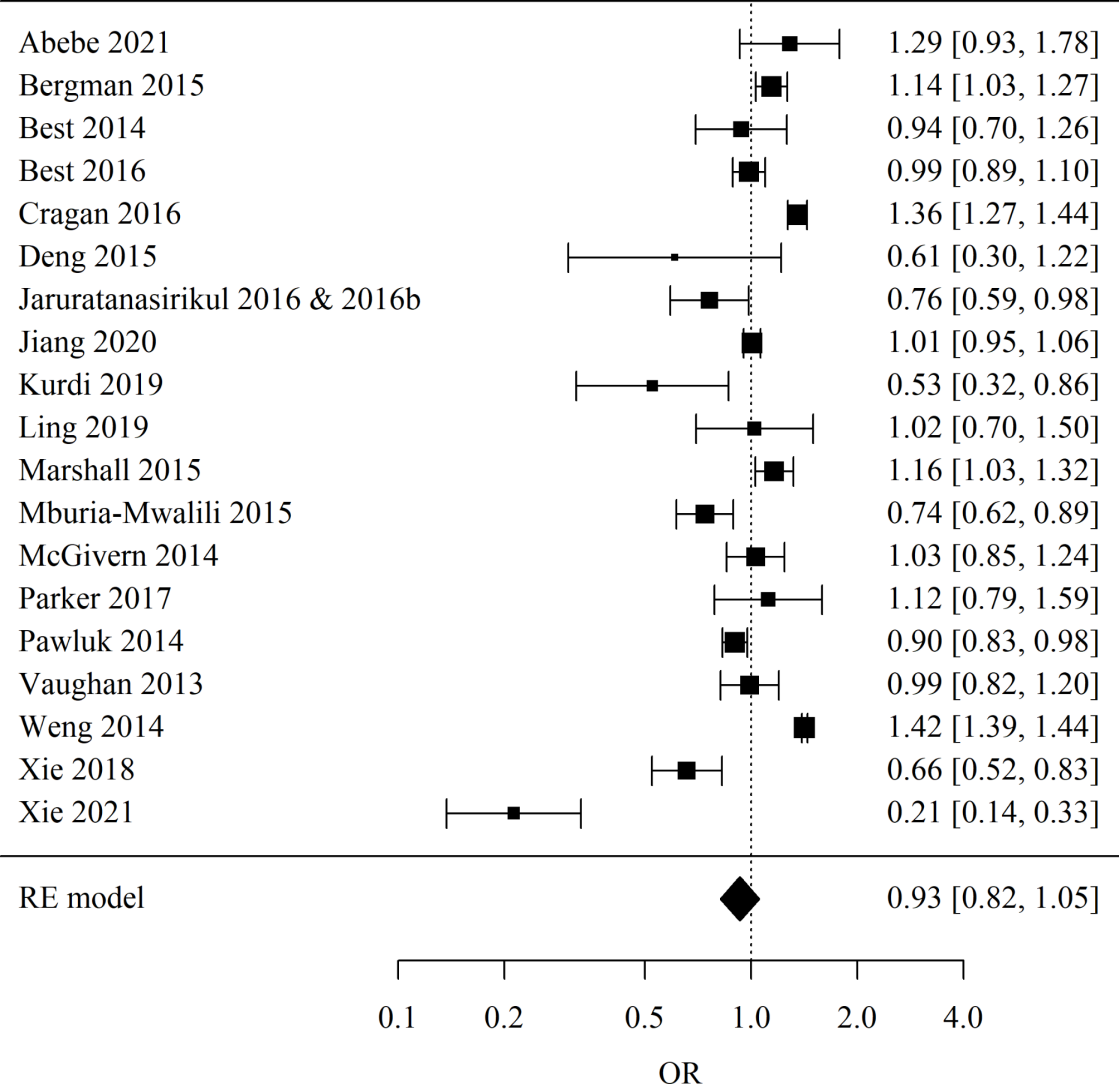
Forest plot for meta-analysis.

The meta-analysis utilized the Odds Ratio (OR) as the chosen effect measure to explore the association between congenital fetal anomalies in children and teenage motherhood. Forest plot for meta-analysis is presented in Fig. 2. The significance level of 0.05 was adopted in the analysis. So, all p-values below 0.05 were interpreted as indicating significant relationships. The individual studies incorporated into the analysis present a spectrum of outcomes. Some studies, such as Weng 2014^25^ and Cragan 2016^12^, report an OR greater than 1, indicating a higher occurrence of congenital fetal anomalies in children of teenage mothers. On the other hand, Xie 2021^26,27^ suggests a reduced risk, with an OR less than 1. The collective findings, as represented by the pooled OR of 0.93, cross the null effect line (OR of 1). This crossing indicates no statistically significant overall association between the age of the mother and the prevalence of congenital fetal anomalies in their children when considering the combined data from all included studies (*p* = 0.25). A test for heterogeneity revealed significant variation among the studies (*p* < 0.001), prompting the use of a random-effects model to derive the reported results. The heterogeneity coefficient I^2^ was calculated to be 96.21%.

**Fig. 3:**
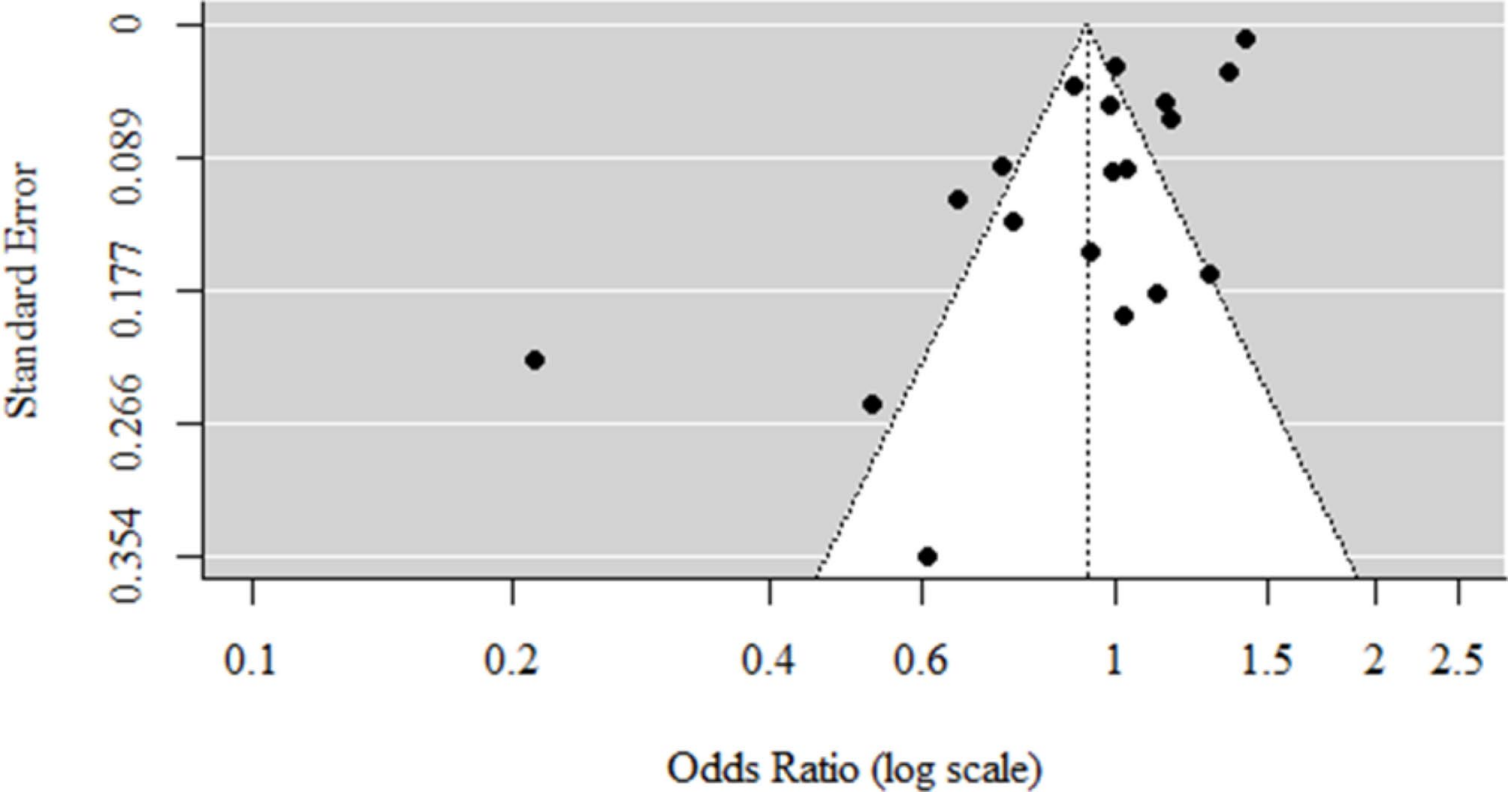
Funnel plot for the meta-analysis.

The funnel plot used for visual representation displayed asymmetry, suggesting the presence of publication bias. To confirm this observation formally, an Egger’s test was conducted, yielding a p-value of < 0.001, thereby establishing significant asymmetry in the plot. Further examination of the plot reveals that its asymmetry is primarily attributed to the lack of studies in the lower right corner. The lower right corner of the plot represents studies in which the percentage of anomalies among teenagers was higher than among non-teenagers (OR > 1), accompanied by a large standard error for this estimation. A large standard error typically indicates smaller studies or studies in which the analyzed phenomenon occurred infrequently (potentially due to the adopted definition of “anomaly”). This means there is a high likelihood that smaller studies with negative or null results are underrepresented in the literature, possibly because such studies are less likely to be published. (Fig. 3)

The meta-analysis did not find statistically significant differences in the occurrence of congenital fetal anomalies between fetuses of teenagers and non-teenagers, with OR 0.93 (95% CI: 0.82–1.05, *p* = 0.25). However, substantial heterogeneity was observed among the included studies, and there was strong evidence of publication bias, particularly related to studies in the lower right corner of the funnel plot. These findings suggest that caution should be exercised when interpreting the results, and further investigation may be warranted to understand the reasons behind the publication bias.

#### Sub-group analysis

The meta-analysis was performed in sub-groups. Dividing the publications into the geographical origin and classification of anomalies depending on the system they affect.

##### Sub-group depending on geographical origin

**Fig. 4:**
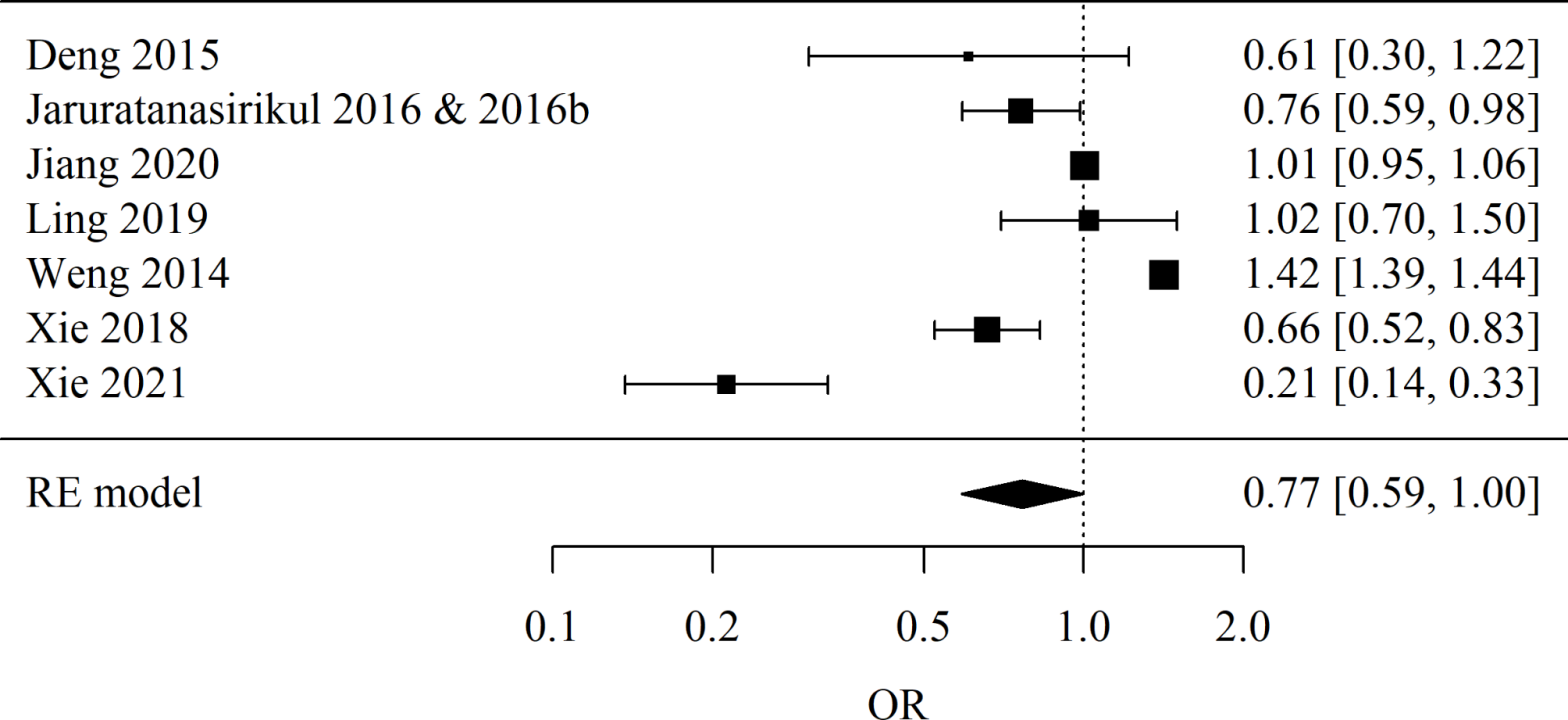
Forest plot for sub-group Asia meta-analysis.

Asia: Number of studies included in the meta-analysis: 7. The Odds Ratio (OR) was used. The result of the meta-analysis is statistically significant (*p* = 0.047), indicating evidence of significant differences between teenagers and non-teenagers. OR = 0.77, meaning that teenage age reduces the chances of defects by 23.4%. The heterogeneity test showed significant heterogeneity of the studies (*p* < 0.001), so the above results were obtained from a random-effects model. The heterogeneity coefficient I² was 97.78%. Forest plot for sub-group Asia meta-analysis is presented in Fig. 4.

**Fig. 5:**
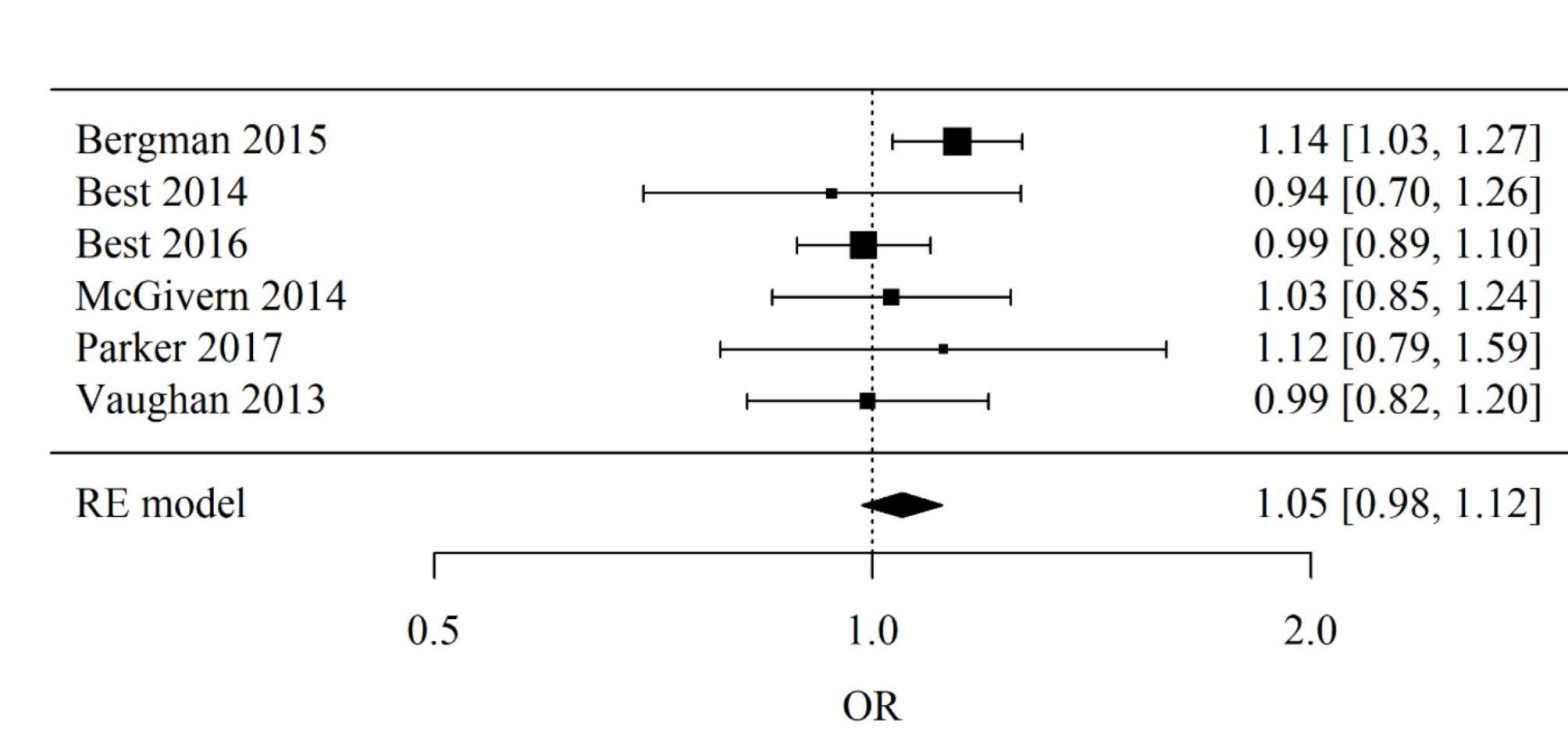
Forest plot for sub-group Europe meta-analysis.

Europe: Number of studies included in the meta-analysis: 6. The Odds Ratio (OR) was used. The result of the meta-analysis is not statistically significant (*p* = 0.144), indicating no evidence of significant differences between teenagers and non-teenagers. The heterogeneity test showed no significant heterogeneity of the studies (*p* = 0.405), so the above results were obtained from a fixed-effects model. The heterogeneity coefficient I² was 1.72%. Forest plot for sub-group Europe meta-analysis is presented in Fig. 5.

**Fig. 6:**
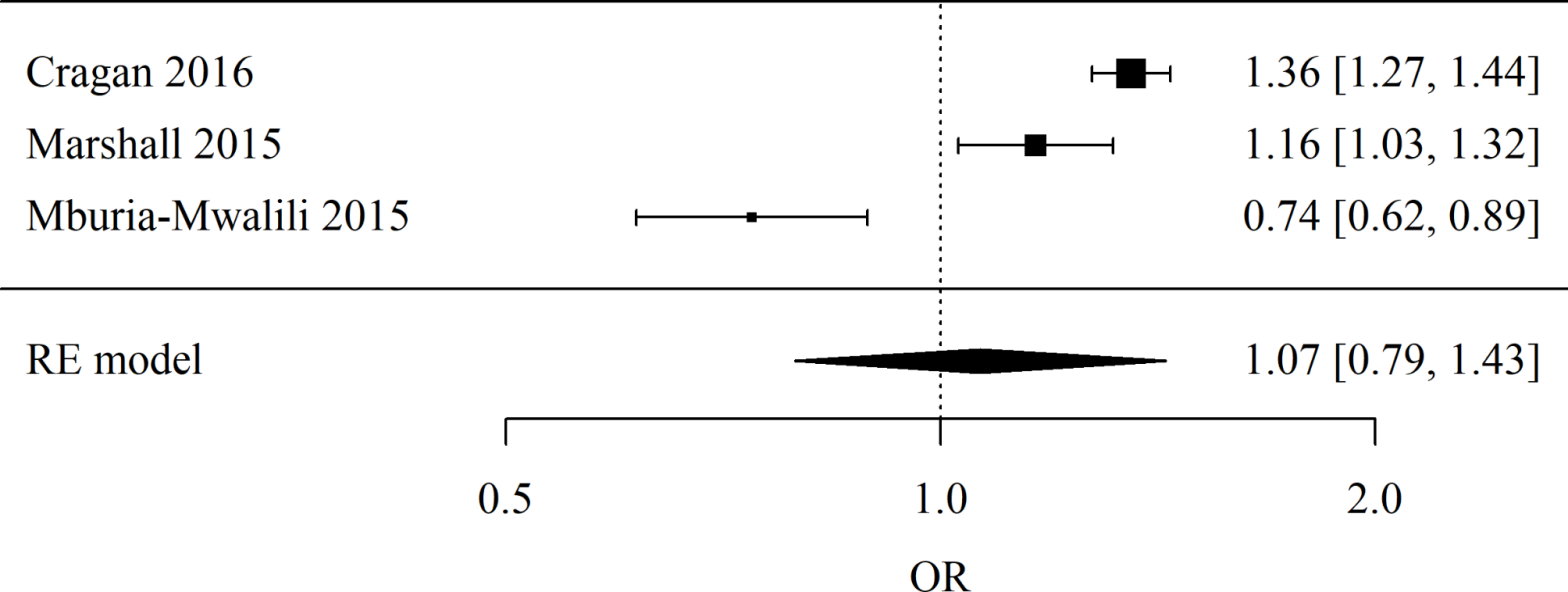
Forest plot for sub-group USA meta-analysis.

Unites states of America: Number of studies included in the meta-analysis: 3. The Odds Ratio (OR) was used. The result of the meta-analysis is not statistically significant (*p* = 0.675), indicating no evidence of significant differences between teenagers and non-teenagers. The heterogeneity test showed significant heterogeneity of the studies (*p* < 0.001), so the above results were obtained from a random-effects model. The heterogeneity coefficient I² was 94.81%. Forest plot for sub-group USA meta-analysis is presented in Fig. 6.

The analysis conducted as part of the meta-analysis for different regions – Asia, Europe, and the USA – yielded intriguing results regarding the relationship between teenage age and the occurrence of congenital fetal anomalies.

In Asia, among the 7 included studies, a statistically significant association (*p* = 0.047) was observed between teenage age and the reduced risk of congenital fetal anomalies, with an OR coefficient of 0.77, indicating a 23.4% decrease in the chances of these defects. It is notable that despite the availability of legal and accessible abortions in the countries included in the meta-analysis, such as China, Thailand, and Taiwan, the abortion rate is significantly higher than in European countries and the USA^28^.

In Europe, despite including 6 studies, no statistically significant differences (*p* = 0.144) were noted between teenagers and non-teenagers regarding the occurrence of congenital fetal anomalies. The lack of significant heterogeneity in the studies (*p* = 0.405) suggests some consistency in the results, possibly due to similar healthcare and social practices in the studied populations.

In the USA, among the 3 studies included, no statistically significant differences (*p* = 0.675) were found between teenage age and the occurrence of congenital fetal anomalies. However, there is significant heterogeneity in these studies (*p* < 0.001), indicating potential differences in research methodology or populations.

##### Sub-groups based on congenital fetal anomalies according to the system they affect

**Fig. 7:**
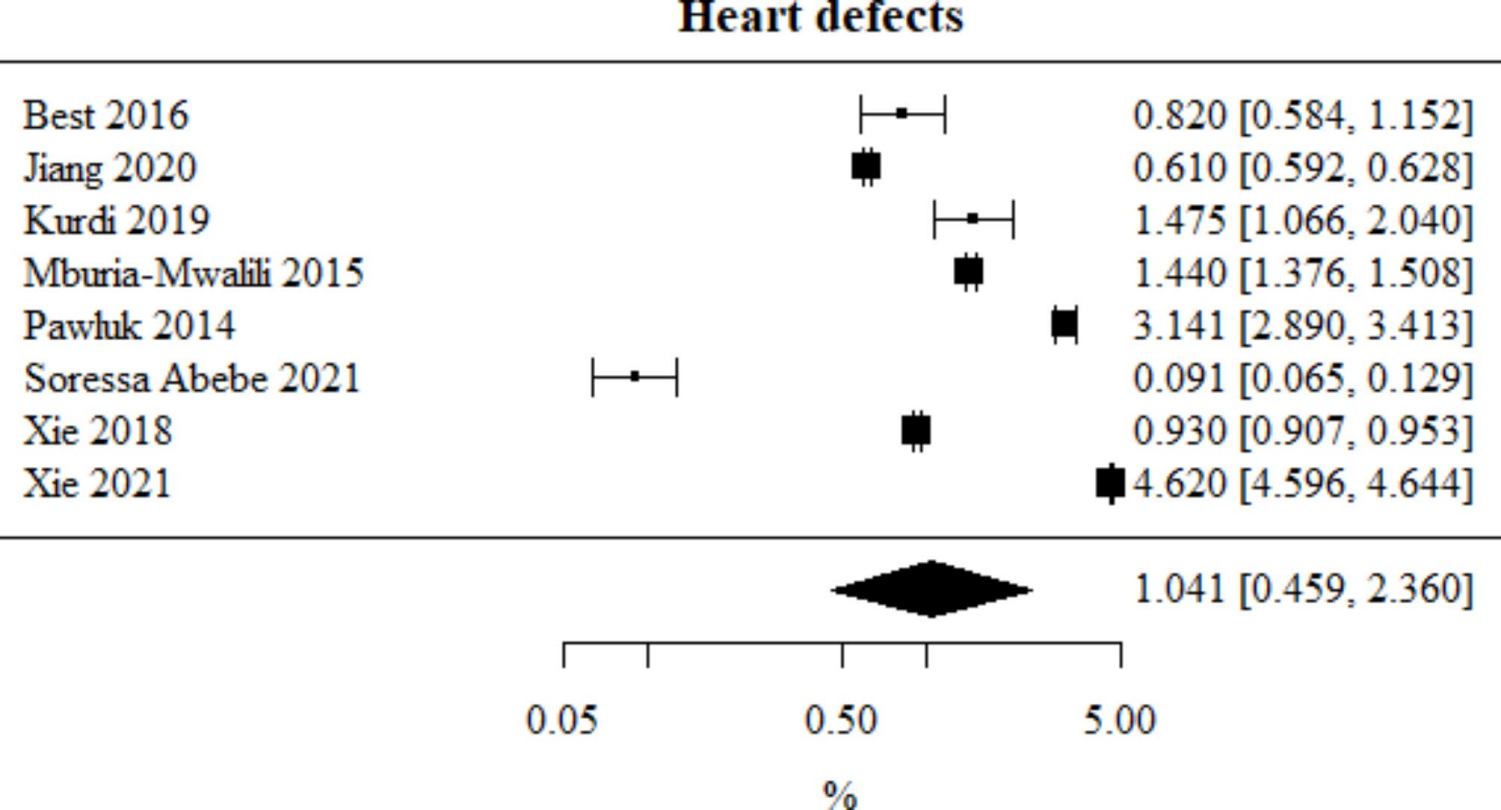
Forest plot for heart defects sub-group meta-analysis.

Heart defects: Number of studies included in the meta-analysis: 8. The percentage of this type of defect, obtained from the combined study results, was 1.041%. The heterogeneity test showed significant heterogeneity among the studies (*p* < 0.001), hence the above results were obtained using a random-effects model. The heterogeneity coefficient I^2^ was 99.98%. Heart defects meta-analysis is presented in Fig. 7.

**Fig. 8:**
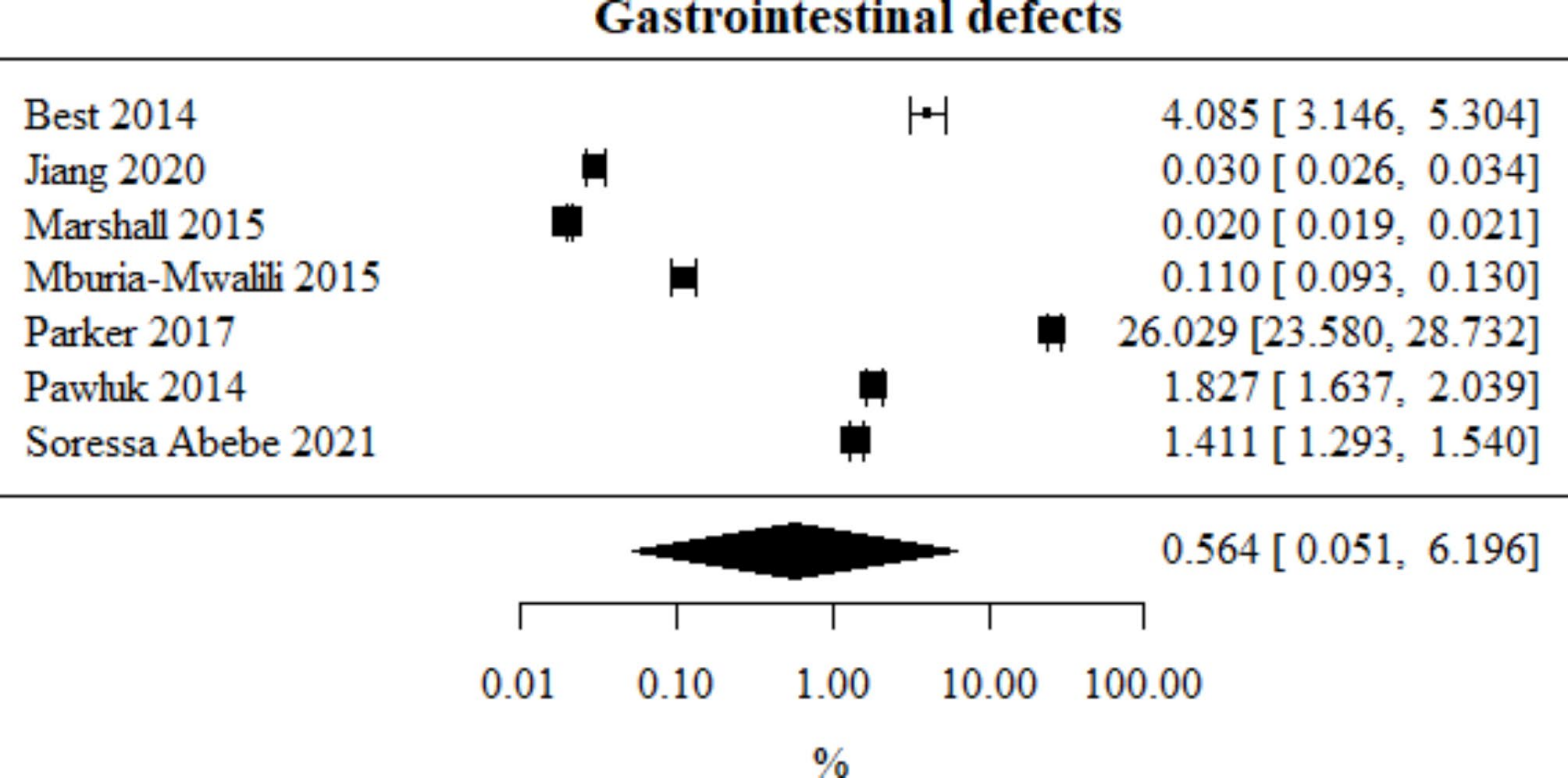
Forest plot for gastrointestinal defects sub-group meta-analysis.

Gastrointestinal defects: Number of studies included in the meta-analysis: 7. The percentage of this type of defect, obtained from the combined study results, was 00.564%. The heterogeneity test showed significant heterogeneity among the studies (*p* < 0.001), hence the above results were obtained using a random-effects model. The heterogeneity coefficient I² was 99.98%. Special handling was required for studies like Pawluk’s, where multiple defect rates were combined to avoid over-weighting. Gastrointestinal defects meta-analysis is presented in Fig. 8.

**Fig. 9:**
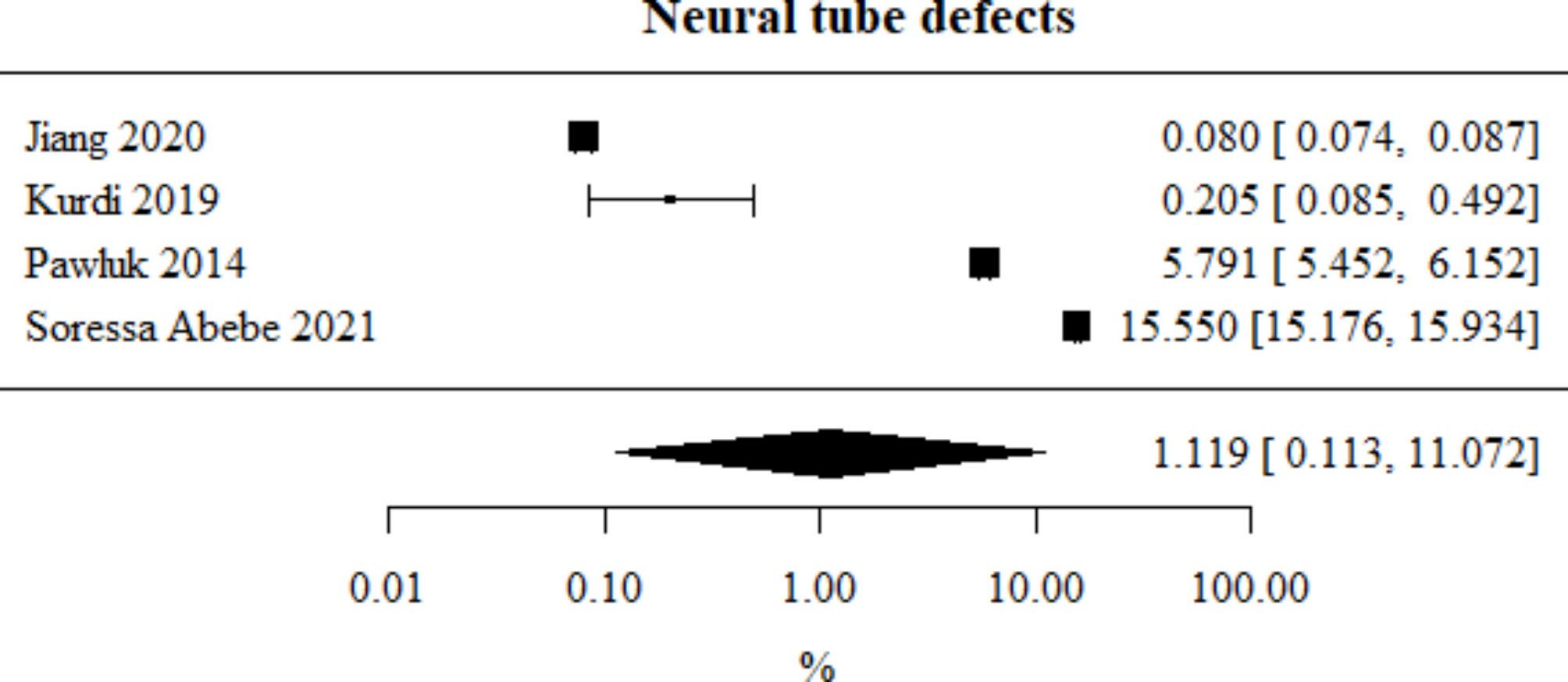
Forest plot for neural tube defects sub-group meta-analysis.

Neural tube defects: Number of studies included in the meta-analysis: 4. The percentage of this type of defect, obtained from the combined study results, was 1.119%. The heterogeneity test showed significant heterogeneity among the studies (*p* < 0.001), hence the above results were obtained using a random-effects model. The heterogeneity coefficient I² was 99.98%. Neural tube defects meta-analysis is presented in Fig. 9.

**Fig. 10:**
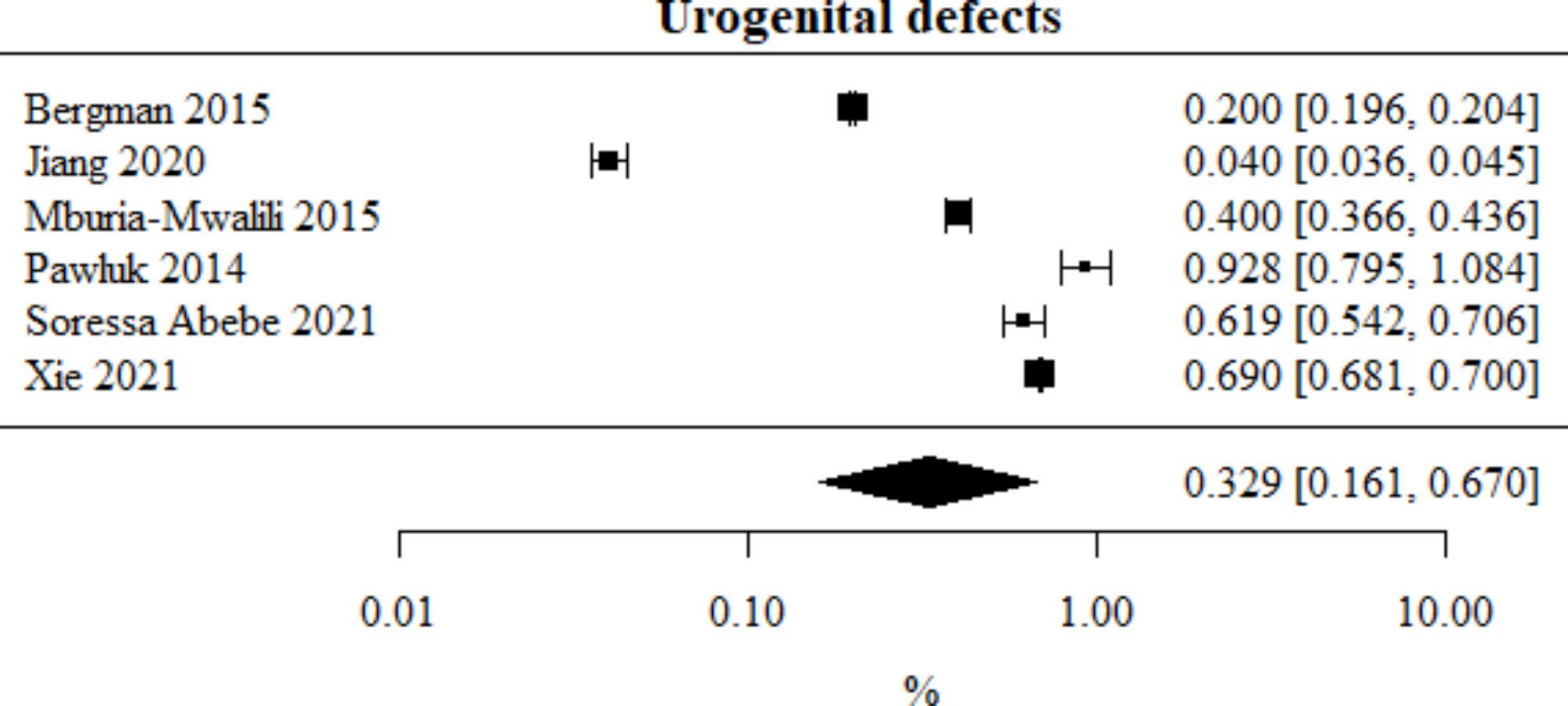
Forest plot for urogenital defects sub-group meta-analysis.

Urogenital defects: Number of studies included in the meta-analysis: 6. The percentage of this type of defect, obtained from the combined study results, was 0.329%. The heterogeneity test showed significant heterogeneity among the studies (*p* < 0.001), hence the above results were obtained using a random-effects model. The heterogeneity coefficient I² was 99.96%. Urogenital defects meta-analysis is presented in Fig. 10.

**Fig. 11:**
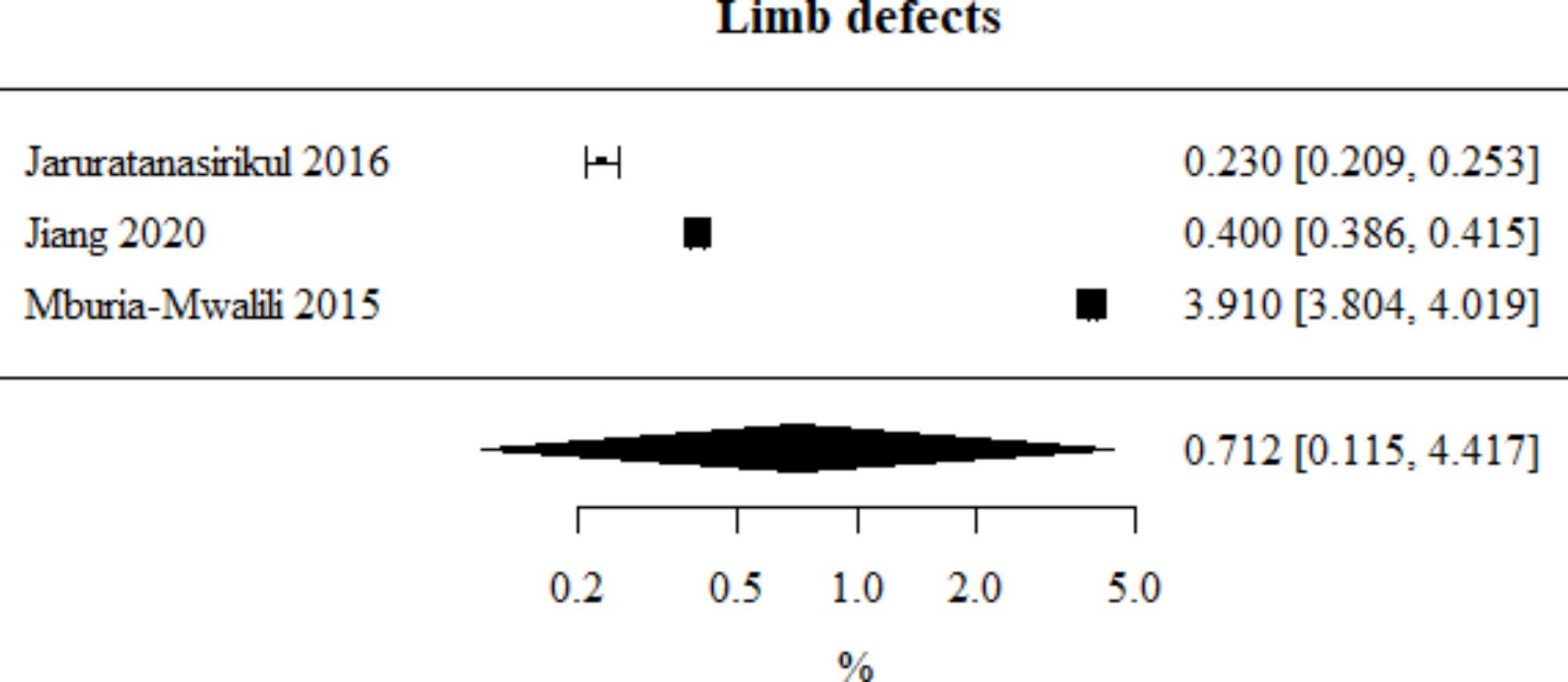
Forest plot for limb defects sub-group meta-analysis.

Limb defects: Number of studies included in the meta-analysis: 3. The percentage of this type of defect, obtained from the combined study results, was 0.712%. The heterogeneity test showed significant heterogeneity among the studies (*p* < 0.001), hence the above results were obtained using a random-effects model. The heterogeneity coefficient I² was 99.98%. Limb defects meta-analysis is presented in Fig. 11.

**Fig. 12:**
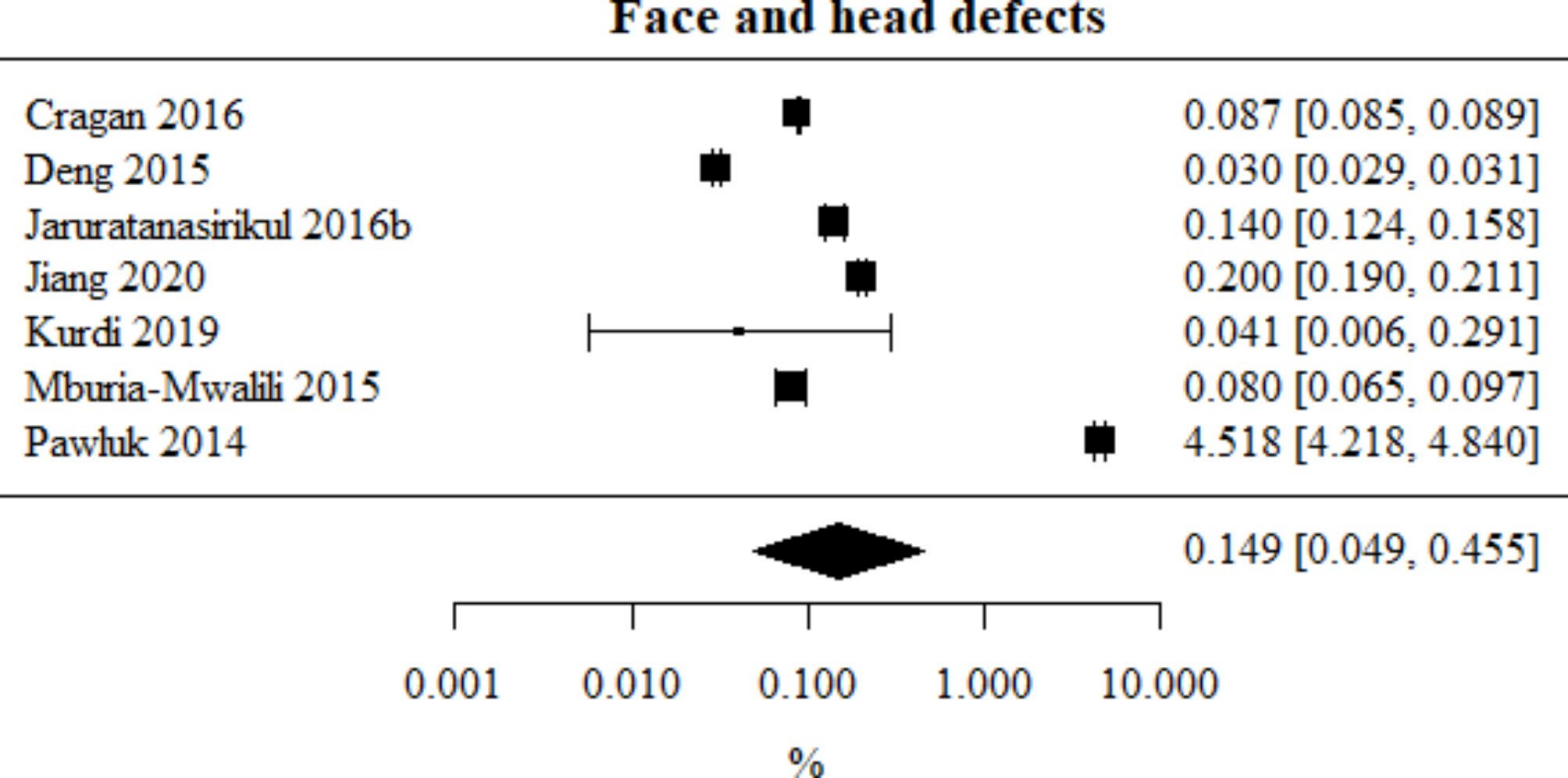
Forest plot for face and head defects sub-group meta-analysis.

Face and head defects: Number of studies included in the meta-analysis: 7. The percentage of this type of defect, obtained from the combined study results, was 0.149%. The heterogeneity test showed significant heterogeneity among the studies (*p* < 0.001), hence the above results were obtained using a random-effects model. The heterogeneity coefficient I² was 99.96%. The face and head defects meta-analysis is presented in Fig. 12.

The analysis consistently indicated significant heterogeneity (*p* < 0.001) with I² values near 100% for all defect types, suggesting that differences between study results are primarily due to factors other than random chance. These factors could include variations in study designs, diagnostic criteria, population characteristics, and environmental and genetic factors. The use of random-effects models was essential to obtain reliable combined prevalence estimates, accounting for the observed variability. However, the high I² values highlight the need for careful interpretation and suggest further research to understand the underlying causes of this variability.

## Discussion

The included studies demonstrate a range of odds ratios (OR) and relative risks (RR), quantifying the probability of fetal abnormalities in younger mothers (≤ 20 years) compared to their older counterparts (> 20 years). For example, Soressa Abebe’s 2021 study^8^ in Ethiopia reported an OR of 1.28, indicating a marginally increased risk in younger mothers. Conversely, Deng’s 2015 study^13^ in China found a reduced risk (OR: 0.61). This compilation of research, including works by Soressa Abebe^8^, Bergman^9^, Best^10,11^, and others, presents a wide array of data from regions such as Ethiopia, Europe, the US, China, Thailand, and Saudi Arabia, offering varied ORs and RRs for fetal abnormalities in younger versus older mothers. This indicates that the association between young maternal age and fetal abnormalities is a global concern, with significant regional variations.

Deng’s study^12^ in China reports a lower prevalence of anotia/microtia, contrasting with Jaruratanasirikul’s findings^14^ in Thailand, which indicate higher rates of congenital limb defects. These geographical variations suggest that environmental and genetic factors may interact with maternal age to influence the risk of fetal abnormalities. Significant regional variations are noted in the prevalence rates of fetal abnormalities across different maternal age groups. Notably, higher prevalence rates are observed in younger mothers in some regions, like Ethiopia (25.6%), in contrast to lower rates reported in Europe and the USA. Additionally, confounding factors in countries where termination of pregnancy is legally accepted may contribute to lower reported prevalence and incidence rates in these countries, as the suspicion of fetal abnormalities during prenatal periods could legally lead to termination of pregnancy. Bergman’s 2015 research^9^ sheds light on the epidemiology of hypospadias in Europe, showing a relative risk of 1.12 in younger mothers. Similarly, Cragan’s 2016 study^12^ in the USA indicates a higher odds ratio (OR: 1.36) for fetal abnormalities among younger mothers. Additional studies from Thailand, Jaruratanasirikul^14,15^, and other areas provide a spectrum of risks, with some suggesting decreased risks in younger mothers.

Several studies, such as Jiang^16^ and Kurdi^17^, highlight the importance of considering additional risk factors, including preconception health and socioeconomic status, in understanding the relationship between young maternal age and fetal abnormalities. The high prevalence of gastroschisis reported by Pawluk et al.^23^ and Parker et al.^22^ shed light on the role of adverse social determinants and environmental exposures, such as maternal infections, in the risk of congenital fetal anomalies. A study by Salihu et al.^29^ found that maternal age younger than 20 years is significantly associated with an increased risk of gastroschisis, with the odds ratio (OR) for mothers aged ≤ 20 years being 7.0 compared to older mothers. Lam et al.^30^ corroborates these findings, reporting that teenage pregnancies have a 5 to 10 times higher risk of gastroschisis compared to pregnancies in older women. Similar conclusions were reached by Feldkamp et al.^31^ and Loane et al.^31^. Loane et al.^32^ found that teenage mothers are six times more likely to have a baby with gastroschisis and nearly five times more likely to experience malformations resulting from maternal infections. Maternal infection, particularly sexually transmitted infections, has also been identified as a risk factor for gastroschisis^33,34^. This risk is further increased when combined with a lower maternal body mass index (BMI). The increased risk associated with low maternal age and pre-pregnancy BMI suggests a potential etiological role related to biological immaturity in the development of this birth defect^35^. However, the specific relationship between maternal age and the risk of gastroschisis remains an area of ongoing research. Although etiological studies have indicated social deprivation, substance abuse, smoking, and low BMI as risk factors for gastroschisis, a comprehensive explanation for the heightened risk associated with young maternal age has yet to be determined^32^. These findings highlight the need for comprehensive public health interventions that address broader socioeconomic and environmental factors. The issue of congenital infection and fetal anomalies in young women is frequently associated with cytomegalovirus (CMV). Primary CMV infections during pregnancy are more likely to occur in younger women, particularly those who have already had at least one child. However, it is important to note that seroprevalence of CMV increases with age and is higher among individuals of lower socioeconomic status, a trend observed in both high-income and low-to-middle-income countries^36^.

Studies show that the incidence of aneuploidy, such as trisomies, is lower in younger age groups. Cuckle et al.^37^ and Hassold et al.^38^ found that the risk of trisomy 21 (Down syndrome), trisomy 18 (Edwards syndrome), and trisomy 13 (Patau syndrome) increases with maternal age, peaking in women over 35 years old. The lower prevalence of chromosomal anomalies in young women contributes to a generally lower incidence of fetal abnormalities in this demographic. In the young maternal population, the risk of these aneuploidies is significantly lower, which may partially explain our findings. Although our study did not find a statistically significant association between young maternal age and the overall incidence of fetal anomalies, it is important to consider other factors. Loane et al. indicated that the risk of non-chromosomal anomalies is age-specific and may vary across different age groups. For example, younger mothers may have a higher risk of certain types of anomalies, while others may be more common in older age groups. *The study by Loane et al. investigated the risk of non-chromosomal anomalies (NCAs) based on maternal age*,* using data from EUROCAT congenital anomaly registers covering 1.75 million births across 23 European regions. The findings indicated that teenage mothers (< 20 years) had a higher risk of NCAs compared to mothers aged 25–29*,* with a relative risk (RR) of 1.11.*^32^. For instance, Hoffman et al.^39^ emphasized the impact of healthcare access on the prevalence of congenital heart disease, which could similarly affect the detection and reporting of other fetal anomalies. These factors include socioeconomic status, access to quality healthcare, and regional disparities.

The observation that primary studies show significant differences, which diminish in collective analysis, suggests that factors beyond age may be influential. Specifically, the limited access to or quality of healthcare in certain regions could be a more critical factor than maternal age itself. The disparities in healthcare access and quality across different regions could significantly influence the incidence of fetal abnormalities. For instance, in areas with limited healthcare resources, younger mothers might not receive adequate prenatal care, leading to a higher prevalence of fetal abnormalities. This scenario contrasts with regions where comprehensive services are more readily available, potentially resulting in lower incidence rates among the same demographic^40–45^. From the physiological perspective, younger mothers often face unique challenges that may contribute to an increased risk of fetal abnormalities. Firstly, adolescents frequently lack access to, or resources for, proper healthcare and prenatal services, factors crucial in monitoring and addressing potential complications during pregnancy. Additionally, it is worth noting that pregnancy for a young woman becomes a physical and psychological burden. Moreover, underage pregnant women may encounter issues such as housing problems or abandonment by their partner. All these factors may result in a lack of proper prenatal care, as adolescent patients may not visit the doctor early enough^4,30,32,46,47^. Nonetheless, none of the analyzed studies reported specifically whether this could have played a role in the findings synthesized in this meta-analysis.

Notably, the studies included in this review exhibited considerable heterogeneity, and there was evident publication bias, especially in studies represented in the lower right quadrant of the funnel plot. These observations necessitate a cautious approach in interpreting the results and underscore the need for further research to elucidate the underlying causes of this publication bias. The aggregate results of the meta-analysis do not reveal statistically significant differences between teenage and non-teenage mothers in terms of fetal anomalies (*p* = 0.252). However, the pronounced heterogeneity among the studies (I² = 96.21%) and the detected publication bias call for a prudent interpretation of these findings. Despite the absence of a statistically significant link between young maternal age and fetal abnormalities, the variation in outcomes across individual studies and the observed publication bias highlight the complexity of this topic. Healthcare professionals are advised to consider these findings within the scope of individual patient care and broader public health initiatives, emphasizing the need for comprehensive prenatal care and counseling for expectant mothers^4,48^.

In summary, this systematic review and meta-analysis offer valuable insights but also emphasize the intricate nature of the relationship between young maternal age and fetal abnormalities. The findings suggest that while young maternal age alone may not be a significant risk factor, the notable heterogeneity and publication bias in the existing literature necessitate careful interpretation, underscoring the need for further research.

## Limitations

Despite adopting a structured approach to the analysis, it is crucial to acknowledge potential limitations within the manuscript. Firstly, the studies included exhibit significant heterogeneity, spanning a broad spectrum of geographical locations, and encompassing demographically diverse populations. This diversity could introduce variability in the findings. Additionally, in some countries, the real incidence of fetal malformations reported postnatally may be underrepresented due to the legality of pregnancy terminations based on prenatal diagnosis, which could skew the data on congenital fetal anomalies. The variability in abortion laws across countries can significantly affect the reported incidence of fetal abnormalities. In regions where abortion is legal and accessible, pregnancies with diagnosed fetal anomalies may be more likely to be terminated, potentially leading to lower reported rates of such anomalies at birth. Conversely, in countries with restrictive abortion laws, higher rates of fetal abnormalities may be reported postnatally. This legal landscape must be considered when interpreting the prevalence and incidence of congenital fetal anomalies in global research. Importantly, socioeconomic factors, the capabilities of healthcare systems, cultural practices, and the public health strategies implemented in each specific country might also influence the observed outcomes. The inclusion of various study designs, including case-control, cohort, and cross-sectional studies, introduces further variability in the quality and reliability of the evidence presented. Moreover, while some studies may focus on specific anomalies, others examine a broader spectrum of fetal anomalies, adding to the complexity of synthesizing the data. Lastly, despite the analysis covering studies conducted over durations ranging from 2014 to 2021, the rapidly evolving landscape of healthcare practices and advancements in prenatal care may not be fully captured within the selected studies. These advancements could significantly impact the incidence and detection of fetal abnormalities, suggesting that the findings might not entirely reflect the current state of prenatal care and its outcomes.

## Implications

While the meta-analysis does not find a statistically significant correlation between young maternal age and fetal abnormalities, the variability in individual study results and the presence of publication bias highlight the complexity of this issue. These findings suggest that caution should be exercised when interpreting the results, and further investigation may be warranted to understand the reasons behind the publication bias. The observation that primary studies show significant differences, which diminish in collective analysis, suggests that factors beyond age may be influential. Specifically, the limited access to or quality of healthcare in certain regions could be a more critical factor than maternal age itself. Healthcare providers should consider these findings in the context of individual patient care and broader public health strategies. It underscores the importance of comprehensive prenatal care and counseling for all expectant mothers, regardless of age.

## Electronic supplementary material

Below is the link to the electronic supplementary material.

Supplementary Material 1

## Data Availability

All data generated or analysed during this study are included in this published article.
